# Le colobome de la paupière supérieure

**DOI:** 10.11604/pamj.2014.17.132.4006

**Published:** 2014-02-25

**Authors:** Hakima Elouarradi, Rajae Daoudi

**Affiliations:** 1 Université Mohammed V Souissi, Service d'Ophtalmologie A de l'Hôpital des Spécialités, Centre Hospitalier Universitaire, Rabat, Maroc

**Keywords:** Colobome, paupière, syndromes malformatifs, Coloboma, eyelid, malformation syndromes

## Image en medicine

Nouveau né de 3 mois, présente un colobome unilatéral isolé du tiers de la paupière supérieure droite (A,B). L'examen général est normal. Un traitement médical à base de lubrifiants en collyre et de pansement oculaire le soir en attendant l'acte chirurgical est instauré. La reconstruction chirurgicale à l’âge de 4 mois a consisté en un simple rapprochement bord à bord, après avivement économique des berges et suture en trois plans selon la règle des quarts de Mustardé (C). Les résultats post opératoires esthétiques et fonctionnels sont satisfaisants (D). Le colobomes palpébral est une affection congénitale rare, souvent isolé ou parfois associé à des syndromes malformatifs (des fentes orbito maxillaires). Le défect peut être partiel ou de pleine épaisseur, localisé souvent au niveau de la moitié médiale de la paupière supérieure ou la moitié latérale de la paupière inférieure. Sa dimension varie d'une simple fissure de la marge palpébrale à un large défect triangulaire ou quadrilatéral ou même absence totale de la paupière. Sa prise en charge doit être précoce vers l’âge de 3 mois, permettant la protection cornéenne, par une réparation du défect palpébral et des anomalies oculaires associées. La décision thérapeutique dépend de la dimension du défect palpébral. Les petits défects de 30% ou moins de la longueur totale de la paupière, peuvent être simplement suturés directement après avivement des berges. Les défects entre 30% et 50% de la longueur palpébrale nécessitent un lambeau semi circulaire de Tenzel avec cantholyse latérale relaxante. Les colobomes plus importants nécessitent une reconstruction palpébrale majeure.

**Figure 1:**
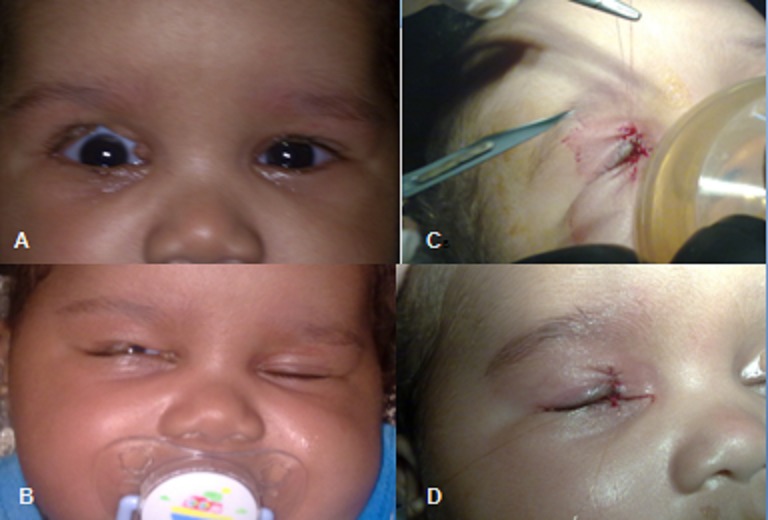
(A,B) Aspect d'un colobome unilatéral isolé de la paupière supérieure droite. (C,D) Aspect per et postopératoire immédiat de la reconstruction chirurgicale

